# A Surprising Finding after External Ear Polypectomy in a Deaf Mute Patient

**DOI:** 10.1155/2015/401708

**Published:** 2015-02-28

**Authors:** Hazem M. Abdel Tawab, Ravi Kumar V, Salim M. Sloma Tabook

**Affiliations:** ^1^Department of Otorhinolaryngology, Sultan Qaboos Hospital, Salalah, Oman; ^2^Department of Otorhinolaryngology, Faculty of Medicine, Cairo University, Egypt

## Abstract

*Introduction*. External auditory canal polyps usually reflect an inflammatory process. Rarely, they may reflect a serious condition that warrants urgent intervention. *Case Report*. A 19-year-old deaf mute female presented to our department with persistent left ear discharge and a reddish mass in the ear. After surgery, the cause was identified as a neglected foreign body. Tympanic membrane was intact. *Conclusion*. Aural polyp that is resistant to medical treatment should raise the suspicion of an inflammatory polyp with underlying chronic suppurative otitis media or foreign body. Rarer neoplastic and immunological causes should also be considered.

## 1. Introduction

Aural polyp or otic polyp is a proliferation of granulation tissue with chronic inflammatory cells in response to a long standing inflammatory process [1, 2]. It is an uncommon lesion, which usually affects young ages, with male to female ratio of 2 : 1 [1–3].

The most common symptoms of an aural polyp are otorhhoea, diminished hearing, and a visible mass in the ear. Otalgia and bleeding or sensation of mass are far less common [2].

Grossly, aural polyp is usually solitary, polypoidal with reddish surface, and often friable [3].

The commonest causes of aural masses are inflammation, cholesteatoma, abscess, benign tumors such as osteomas, and malignant tumors like rhabdomyosarcoma and squamous cell carcinoma [4].

Patients with foreign body might present with a history of foreign body insertion, pain, hearing loss, or otorrhoea or as an incidental finding on clinical examination [5]. Several large case series focusing on children found that 75 percent of patients with ear foreign bodies were younger than eight years [6] while similar studies of adult patients are lacking.

Foreign body presenting as a granuloma or polyp is rare and is found uncommonly in the literature. The present case report emphasizes the need to keep an underlying foreign body in mind when one encounters an aural polyp in clinical practice. This will prevent unnecessary interventions.

## 2. Case Report

A 19-year-old female presented to our department with a history of persistent purulent discharge from the left ear of three-month duration associated with a reddish mass for two months. She is congenitally profoundly deaf with no intelligible speech. She did not have any past history to suggest chronic suppurative otitis media. She denied any ear pain, giddiness, facial weakness, or other symptoms to suggest complications. There was no preceding history of rhinitis. There was no history of dermatitis or seborrhea.

Otoscopy revealed a pinkish polyp completely filling the left ear canal and nonfoul smelling purulent discharge. The exact site of origin could not be established with a probe test. The polyp looked smooth, nonulcerative, and nontender. The right ear, nose, throat, neck, and systemic examination were normal.

A one-week course of amoxicillin clavulanate and topical antibiotic steroid drops was prescribed after cauterization with silver nitrate. This reduced the size of the polyp and the discharge. Two further attempts at cautery failed to give any benefit and the polyp started to grow to its pretreatment size and the discharge resumed. A computed tomography (CT) scan of the temporal bones was requested.

**Figure 1:**
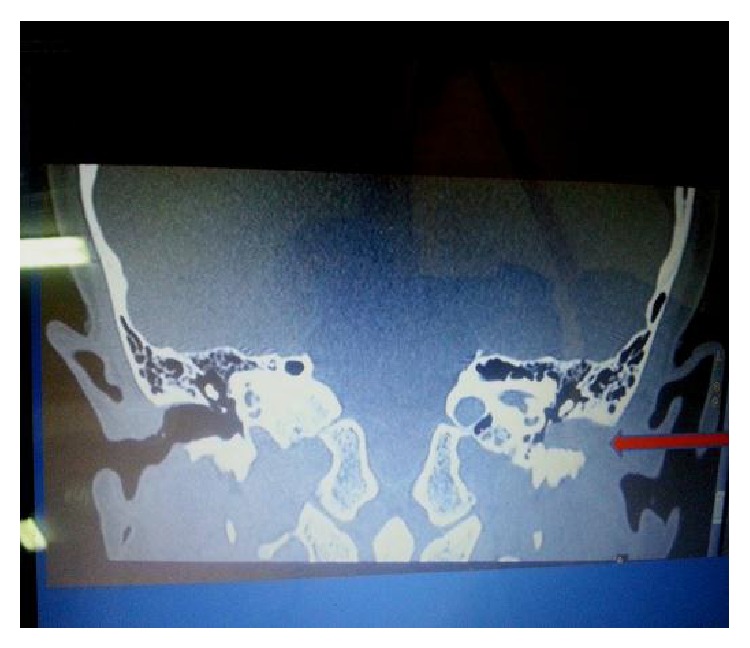
Left aural polyp.

**Figure 2:**
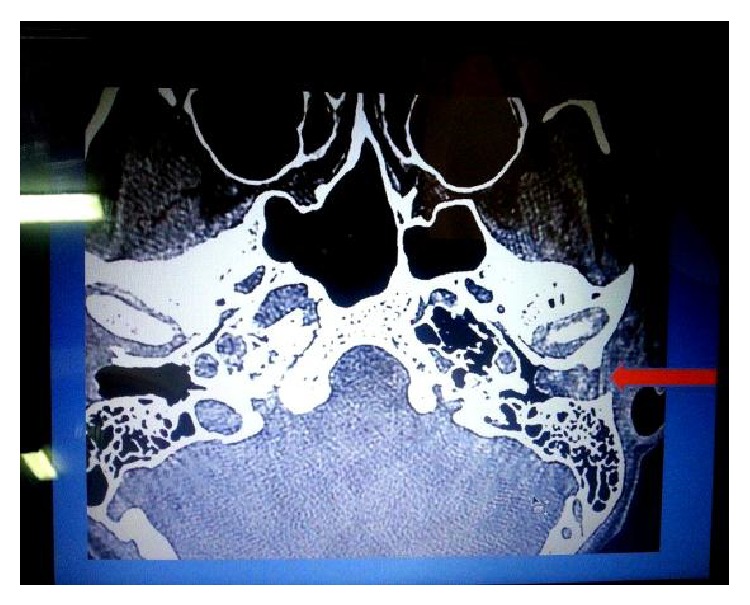
Pneumatized mastoid and middle ear.

The CT scan revealed a soft tissue mass completely filling the left external auditory canal and pushing the left tympanic membrane medially towards the middle ear cavity with normal pneumatization of the mastoid air cells and the rest of the middle ear cavity (Figures 1 and 2).

**Figure 3:**
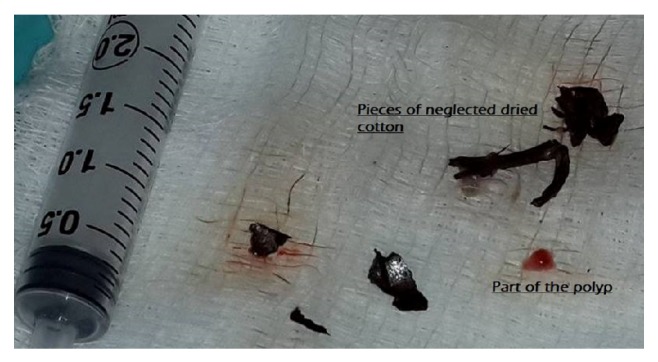
Part of the polyp after excision and foreign body cotton dry pieces.

Informed consent was taken from the patient and patient's guardian after explanation. Left complete aural polypectomy was done under general anesthesia using the operating microscope. The polyp had a broad attachment to the superior and posterior meatal walls and the ear canal skin was excoriated. Dark black material, firm in consistency and friable, was found filling the meats up to the tympanic membrane deep to the polyp. Tympanic membrane was found to be intact. The removed foreign body pieces (Figure 3) appeared to suggest retained cotton. The ear canal was packed with medicated ribbon gauze. When the intraoperative findings were discussed with the patient, she mentioned that it might be a piece of cotton she used for cleaning her left ear. On removal of the pack after two days, the skin was well healed and the patient remained symptom-free at the one-month followup visit.

Histopathological assessment of the specimen revealed an inflammatory epithelial polyp which was polypoidal and covered by squamous epithelium. The epithelium showed hyperkeratosis, parakeratosis, and acanthosis. Stroma showed proliferated capillaries, hemorrhage, and inflammatory cell infiltration. Another area showed ulcerated inflammatory granulation tissue with a focus of foreign body giant cell reaction.

## 3. Discussion

Aural polyps are often attributed only to chronic suppurative otitis media. This case illustrates that other causes should be borne in mind and it is prudent to actively rule out a foreign body by detailed history. When the CT scan rules out middle ear and mastoid involvement, other primary external auditory canal diseases need to be considered [3].

Usually, aural polyps are of inflammatory origin composed of nonspecific inflammatory granulation tissue. These simple aural polyps can offer microscopical clues to some underlying complications of chronic otitis media. Others may reveal clinically unsuspected diseases [3].

In a study done by Gliklich et al., in 1993, they mentioned that 15/35 of their patients with aural polyps were found to have chronic suppurative otitis media and 10/35 had cholesteatoma, while 8 patients had aural polyps on top of foreign body (ventilation tubes) [2].

In his review in 1990, Friedmann stated that the possible causes of aural polyp in the ear might include pyogenic granuloma, relapsing polychondritis, Langerhans cell histiocytosis, neoplastic conditions like osteoma, tumours of the ceruminous glands, rhabdomyosarcoma, and progressive necrotizing otitis externa [3]. Tuberculous middle ear infection can form aural polyp that is formed entirely of tuberculous granulation tissue [7]. Wegener's, sarcoidosis, meningioma [8], and metastatic renal cell carcinoma [9] can also rarely present as aural polyp.

Gentle aural cleansing and application of antibiotic-steroid drops can be helpful in reducing the size of the polyp. Also, application of silver nitrate can be helpful in the initial therapy of the aural polyp [10]. In our case, regular aural toilet and a trial of cautery with silver nitrate were done with no significant improvement.

CT plays an important role in the diagnosis and in specifically the determination of the extent of the disease. It also provides a tool for better evaluation of the surrounding structures and it is considered as the best method to visualize the middle ear when complete occlusion of the external auditory canal (EAC) is the case [11].

In the study case, complete evaluation of the tympanic membrane and extent of the aural polyp was not possible even with microscopic examination in the outpatient clinic. We employed CT scan to assess the extent of the polyp and the condition of the middle ear and mastoid air cells.

Law and Watters in 1997 described a case of a penetrating oral foreign body presenting with an aural polyp. The possibility of a penetrating oral injury should be considered whenever a child's fall is unwitnessed. They documented that an underlying foreign body should be considered in cases where an aural polyp fails to respond to standard therapy [12].

Harris et al. in 2004 described a case of a child with chronic otorrhea for 4 months not responding to topical and systemic antibiotics. When the case was referred to them, a large external canal polyp covering an electrical cap foreign body was discovered. They stated that external auditory canal polyps that fail to respond promptly to conservative medical therapy warrant a computed tomography scan and surgical exploration with biopsy [13].

In our case, history of the patient's condition did not help to know the actual cause of the aural polyp. The CT scan done after failure of medical treatment did not give any indication of an underlying foreign body, though it ruled out chronic suppurative otitis media. Only an aural polypectomy under general anesthesia elucidated the diagnosis when multiple pieces of foreign body were found between the site of the polyp and the tympanic membrane.

Any aural polyp should be evaluated thoroughly by imaging techniques. Polypectomy should be considered to be mindful that it may uncover a serious disease process or even a surprising one like in our presented case.
